# Engineered Extraocular Muscle with Decellularized Tissue and Synthetic Biodegradable Polymers: Design, Properties, and *In Vivo* Studies

**DOI:** 10.1021/acsbiomaterials.5c00073

**Published:** 2025-08-13

**Authors:** Fatma Yülek, Özge Ekin Akdere, Sena Koç Akbayrak, Menemşe Gümüşderelioğlu, Sevil Çaylı, Ebru Alimoğulları, Ayşen Erdem, Meltem Tuncer, Özbeyen Atalay

**Affiliations:** † Department of Ophthalmology, Yıldırım Beyazıt University Faculty of Medicine, Ankara 06031, Turkey; ‡ Department of Bioengineering, Hacettepe University, Ankara 06800, Turkey; § Department of Chemical Engineering, Hacettepe University, Ankara 06800, Turkey; ∥ Graduate School of Science and Engineering, Hacettepe University, Ankara 06800, Turkey; ⊥ Department of Histology, Yıldırım Beyazıt University Faculty of Medicine, Ankara 06031, Turkey; # Department of Physiology, Faculty of Medicine, Hacettepe University, Ankara 06230, Turkey

**Keywords:** extraocular muscle regeneration, extraocular muscle, poly(caprolactone) (PCL), poly(lactic-*co*-glycolic acid) (PLGA), decellularization, electrospinning

## Abstract

The study aims to develop graft materials suitable for treating severe muscle loss and thyroid ophthalmopathy. A new hybrid graft combining poly­(caprolactone) (PCL), poly­(lactic-*co*-glycolic acid) (PLGA), and decellularized bovine extraocular muscle (dEOM) was created. PLGA membranes were formed via solvent casting, and aligned PCL (aPCL) nanofibers were electrospun onto these membranes, resulting in aPCL–PLGA. Lyophilized dEOM was then powdered and deposited onto the aPCL–PLGA membranes through gelation, creating *g*-dEOM/aPCL–PLGA grafts. These three-layer grafts were characterized physically and chemically, and their muscle regeneration capabilities were assessed through *in vitro* and *in vivo* experiments. *In vitro* results showed that the materials supported mouse myoblast cell (C2C12) adhesion and proliferation. For *in vivo* studies, 30 rabbits underwent surgical procedures to create muscle defects, and tissue samples were collected after 15 and 45 days for analysis. Electrophysiological tests and immunohistological studies indicated that both dEOM and the hybrid graft supported the regeneration of extraocular muscles, enhancing surgical efficacy and providing a viable alternative to autografts by promoting regular fiber development over time.

## Graphical abstract

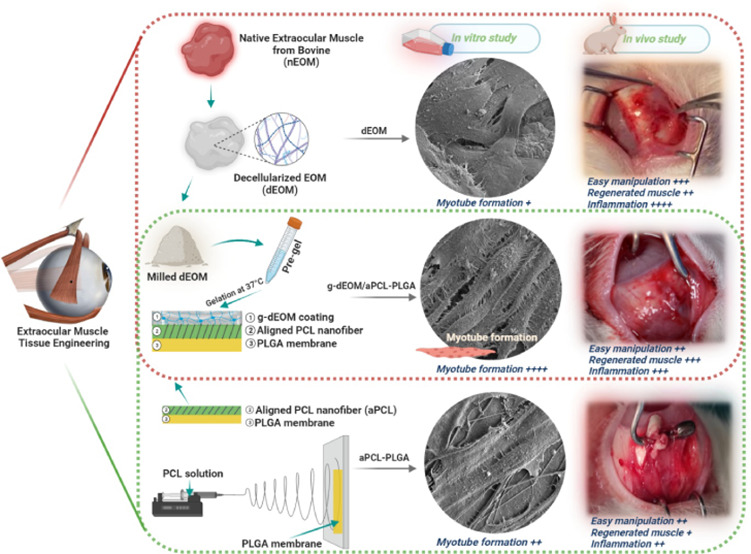

## 1 Introduction

Eye motility problems arising from conditions such as cranial trauma, thyroid eye disease, and recurrent strabismus surgery can lead to significant disabilities and psychosocial challenges for affected individuals.^1^ Extraocular muscles (EOM), responsible for eye movement, possess unique characteristics that distinguish them from other striated muscles. These distinctions include their innervation patterns, histochemical features, ultrastructure,^2^ embryological development,^3^ and the presence of inherent myogenic progenitor cells with varied proliferative and regenerative abilities,^4,5^ as well as intrinsic neurotrophins.^6^ However, extensive injury or fibrosis due to inflammatory disorders, like thyroid eye disease, can result in increased connective tissue scarring and ″tighter″ muscles, altering their functional properties and affecting the conjugation of eye movements.^7,8^

To date, various methods have been explored for closing defects or extending EOM, including autografts,^9,10^ xenografts,^11,12^ and synthetic materials such as silicone. Surgical interventions involving autografts and transposition techniques often fail to fully restore function, leading to anatomical changes, torsional issues, and biomechanical deficiencies in both recipient and donor sites.^9,10,13–15^ Additionally, synthetic materials may provoke severe inflammatory reactions, extrusion, and resistant infections,^16^ while xenogeneic grafts can trigger immunologic responses in this highly vascularized tissue.^12,17^ Lyophilized tissues, such as pericardium and dura mater from porcine and bovine sources, have been utilized in certain clinical scenarios related to scleral issues.^18^ Consequently, improved strategies for the healthy regeneration of EOM are still under investigation.

Tissue engineering has provided numerous options for skeletal muscle regeneration.^19^ In this context, new tissue engineering approaches for EOM regeneration, which has distinct properties compared to other striated muscles, may offer physiological treatment options for eye muscle injuries and degenerative conditions. FDA-approved extracellular matrix (ECM)-based biomaterials derived from decellularized tissues and organs, such as the small intestine, dermis, bladder, and pericardium, have been used for soft tissue repair.^20^ On the other hand, hybrid biomaterials, such as collagen bioactivated electrospun poly­(caprolactone) (PCL) scaffolds, have been used in the regeneration of other striated muscles.^21,22^ Synthetic scaffolds specifically studied for skeletal muscle regeneration include polymers such as PCL, poly­(lactic acid) (PLA), poly­(glycolic acid) (PGA), and their copolymers. However, new treatment approaches for EOM regeneration, which has limited regenerative ability but is more complex than other striated muscles due to its thinness and higher progenitor cell content,^4,5^ have not been fully explored in the light of recent tissue engineering advancements.

We hypothesize that decellularized xenogeneic EOM may be a more effective biomaterial for myoblasts than ECM derived from other tissues (such as dura mater). Based on this information, the present study aims to develop two graft materials for functional EOM repair, as well as for EOM lengthening surgery in challenging clinical situations. The first material involves decellularized bovine EOM. The second material is a hybrid tissue graft designed to create a suitable microenvironment for myoblasts by mimicking EOM architecture. This graft consists of PCL nanofibers aligned on a flexible PLGA membrane, with a powdered decellularized ECM gel on top. The fabrication, characterization, and subsequent *in vitro* and *in vivo* evaluation of these materials will be discussed.

## 2 Materials and Methods

### 2.1 Materials

Poly­(caprolactone) pellets (Mw: 80,000), bovine serum albumin (BSA), 3-[4,5-dimethylthiazol-2-yl]-diphenyltetrazolium bromide (MTT), Ehrlich reagent, glutaraldehyde, hydrochloric acid, isopropanol, chloramine T, lysozyme, pepsin, sodium azide, sodium dodecyl sulfate (SDS), trypsin-EDTA, xylene and trans-4-hydroxy-l-proline were purchased from Sigma-Aldrich (St. Louis, USA). Poly­(lactic-*co*-glycolic acid) (PLGA) (PURASORB PL 18, 50:50 Dl-lactide/glycolide copolymer) was kindly provided from Corbion (Holland). Amphotericin B, chloroform, 1,1,1,3,3,3-hexafluoro-2-propanol (HFIP), eosin Y, hexamethyldisilazane (HMDS), hematoxylin, paraformaldehyde, potassium chloride, sodium bicarbonate, sodium dihydrogen phosphate monohydrate, sodium hydroxide, sodium chloride and Triton X-100 were obtained from Merck (Germany). Horse serum and RPMI were provided from Capricorn Scientific (Germany). 4’,6-Diamidino-2-phenylindole (DAPI) was purchased from Vector Laboratories (California, USA). Ethyl alcohol was provided from Teksol (Turkey). Fetal bovine serum (FBS), l-glutamine and penicillin-streptomycin (P/S) were purchased from Biowest (France). Ketamine hydrochloride injection (Alfamine 10%) and xylazine hydrochloride injection (Alfazyne 2%) were provided from Ege Vet (Turkey). Mallory trichrome was purchased from Bio-Optica (Italy). Dexamethasone disodium phosphate (0.1%) and netilmicin sulfate (0.3%) (Netildex) were provided from Teka (Italy). Polyglactin suture was purchased from Ethicon (USA). Invitrogen Quant-iT PicoGreen dsDNA assay kit was purchased from Thermo Fisher Scientific (USA). The C2C12 cell line was obtained from DSMZ (ACC 565) (Germany).

### 2.2 Fabrication and Characterization of the Grafts

#### 2.2.1 Decellularization of Bovine Extraocular Muscle

Bovine extraocular muscles, isolated immediately after slaughter, were divided into pieces measuring 10 × 10 × 1.5 mm^3^, and the surrounding connective tissue was removed. After washing with phosphate buffer solution (PBS) containing antibiotics (1% v/v, P/S), these muscle pieces were kept in PBS (pH:7.4) prepared with the same antibiotics at 4 °C for 24 h. They were then placed in histological tissue cassettes and treated with 1% (w/v) sodium dodecyl sulfate (SDS)-PBS at 170 rpm on a vortex mixer for 4 days at 24 °C. All other steps were carried out with shaking at room temperature unless stated otherwise. Cassettes containing tissue pieces were washed with ultrapure water for 15 min and kept in 1% Triton X 100 (v/v, prepared with distilled water) solution for 30 min. The tissues were washed again with ultrapure water, kept in ultrapure water for an additional 2 days, and then stored at −80 °C.

Decellularized extraocular muscle (dEOM) and native muscle (nEOM) pieces were evaluated by staining with Hematoxylin and Eosin (H&E) and Mallory Trichrome (MTC), and the sections were examined under a light microscope (Olympus, Tokyo, Japan). For nuclear staining, the sections were covered with 50 μL DAPI (0.1 μg/mL in PBS) and the amount of double-stranded DNA was determined by creating a calibration chart with Picogreen (Quant-iT PicoGreen) according to the manufacturer’s instructions. The total collagen content was determined by hydroxyproline analysis.^23^ The sulfated glycosaminoglycan (GAG) content of the samples was measured using the dimethylmethylene blue dye assay (DMMB) as described in the literature.^24^

#### 2.2.2 Fabrication of Aligned PCL Fibers on PLGA Membranes (aPCL–PLGA)

The grafts were produced by collecting aligned PCL nanofibers on PLGA membranes. The solvent casting method was used to prepare PLGA membranes. PLGA pellets were dissolved in chloroform to obtain a 7% (w/v) PLGA solution, stirred overnight on a magnetic stirrer. The next day, 6 mL of the solution was poured into 7 cm diameter Teflon dishes and allowed to dry at room temperature. Before producing electrospun PCL nanofibers, PCL pellets were dissolved in chloroform:HFIP (2:1 (v/v)) to form a 9% (w/v) PCL solution. The solution was drawn into a 5 mL plastic syringe (Ayset Plastik, Turkey) with a 0.8 mm diameter metal needle and transferred to the electrospinning device (NE Multinozzle Electrospinning System, Inovenso, Turkey). Aligned PCL fibers were collected on PLGA membranes fixed to the rotating cylinder-type collector in the electrospinning device at an optimized rotation speed (2,000 rpm). Electrospinning processes were carried out under the specified conditions (Table S1).

#### 2.2.3 Fabrication of ECM-Coated Hybrid Grafts (*g*-dEOM/aPCL–PLGA)

The decellularized EOM was pulverized by lyophilization (−80 °C, Christ, Germany) and trituration (Retsch MM301, Germany) and finally filtered through a 20 μm filter to remove impurities. A solution consisting of 1 mg/mL pepsin and 10 mg ECM in 0.01 M HCl was prepared. To this ECM solution, 1/9 (v/v) 10× PBS and 1/10 (v/v) 0.1 M NaOH were added, and the solution was diluted to 8 mg/mL by adding 1× PBS. This ECM solution, termed pregel, was poured to cover the 1 × 1 cm^2^ aPCL–PLGA membrane and left to dry for 12 h at 37 °C.

#### 2.2.4 Characterization Studies

The morphology of the grafts was examined by scanning electron microscopy (SEM) (GAIA 3, Tescan Corporation, Czech Republic). Fiber diameters were measured using SEM images with the ImageJ (NIH, USA) software. Furthermore, energy dispersive X-ray (EDX) microanalyzer was used to determine the elemental composition.

Surface wettability of the materials was determined by water contact angle measurements using the sessile drop method (Krüss DSA 100, Germany).

The biodegradability of the grafts was evaluated enzymatically with 10 μg/mL lysozyme in PBS, and the mass loss of grafts was calculated by comparing their initial weights.^25^

The mechanical properties of the grafts were measured with a Stable Micro Systems Texture Analyzer (TA-XT plus, Surrey, UK) in wet state to mimic the body environment.^25^

### 2.3 Cell Culture Studies

Cell culture studies were carried out with mouse myoblast C2C12 cells (ACC 565, DSMZ, Germany) at passage 5 in a laminar flow cabinet (Bioair, Type II Laminar Flow Cabinet, Italy). Cells were subcultured in flasks (Corning, USA) with RPMI containing l-glutamine, 10% (v/v) FBS (fetal bovine serum), and 0.5% (v/v) P/S, and placed in a CO_2_ incubator (Panasonic Instruments, Japan). For cell culture studies, 1 × 1 cm^2^ decellularized bovine eye muscle (dEOM), PCL fiber-coated PLGA membranes (aPCL–PLGA) and ECM gel-coated PCL–PLGA membranes (*g*-dEOM/aPCL–PLGA) were used (Figure 1).

**Figure 1:**
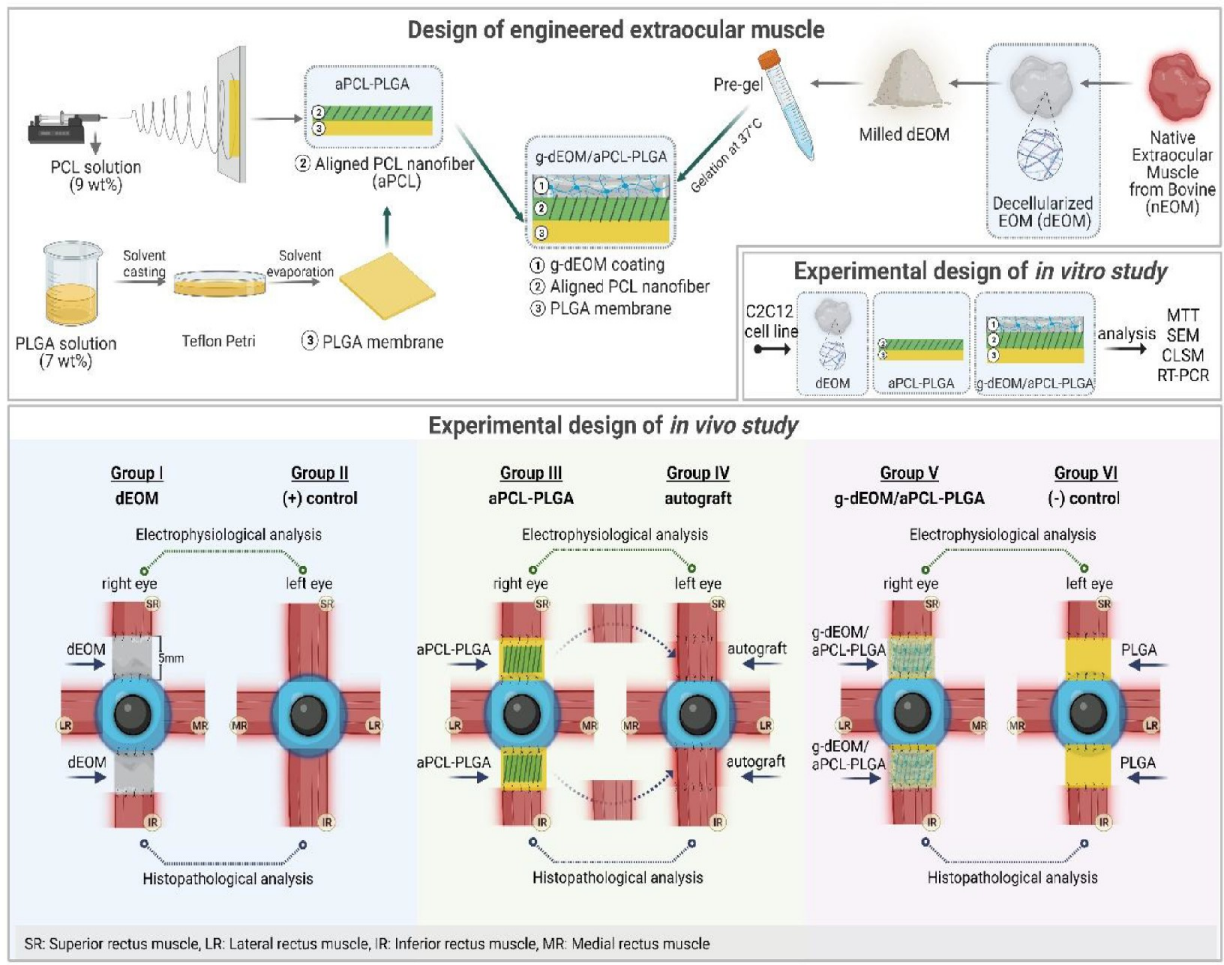
Schematic presentation of engineered extraocular muscle design and groups in *in vitro* and *in vivo* experiments. This diagram was created with BioRender.com.

For sterilization, the grafts were immersed in PBS containing 1% P/S and 0.5% Amphotericin B for 1 day. Following this, the antibiotic solution was removed, and the grafts were allowed to dry in the laminar flow cabinet. To facilitate protein adsorption onto the material surfaces, the grafts were kept in conditioning medium overnight before cell cultivation.

Cell cultivation was conducted statically in 24-well culture dishes. C2C12 cells were seeded onto each graft with 20 μL of growth medium at a density of 2 × 10^5^ cells per graft. To prevent desiccation, 20 μL of growth medium was added to each well hourly, culminating in a total of 1 mL of growth medium after 3 h. On the second day post-transplantation, differentiation medium (RPMI with l-glutamine, containing 2% horse serum and 0.5% P/S) was introduced, and cell culture continued for 14 days, with medium changes occurring twice a week.

Cell viability of C2C12 cells grown on the materials was assessed spectrophotometrically using MTT analysis on various during the culture period.^26^ The morphology and skeletal organization of the cells were visualized via SEM (Tescan, GAIA 3, Czech Republic) on days 3, 7, and 14, and by confocal microscopy (Leica, Germany) on days 7 and 14.^26^ Additionally, F-actin/nucleus staining was performed using Alexa Fluor 488 Phalloidin (1:100) to stain the cytoskeleton and DAPI for nuclear staining.^27^ Imaging was conducted with a confocal microscope.

Differentiation of C2C12 cells was evaluated by reverse transcriptase-polymerase chain reaction (RT-PCR) analysis on days 7 and 14. The expression of differentiation markers MyoD and Myogenin in muscle tissue was analyzed, with β-Actin serving as a control gene (Table S2). Relative expression of the target genes was normalized to β-Actin and calculated using the 2^–ΔΔCt^ method. For RT-PCR analysis, RNA was isolated using the Trizol method and Qiagen’s RNeasy Mini Kit on specified days of culture. cDNA synthesis was performed using the High-Capacity cDNA Reverse Transcription Kit (Applied Biosystems) according to the manufacturer’s protocol. Analysis was conducted on a LightCycler Nano (Roche, Switzerland) using Solis BioDyne’s 5x HOT FIREPol and EvaGreen qPCR Mix Plus (Estonia) kits.

### 2.4 *In Vivo* Animal Experiments

#### 2.4.1 Preparation of Experimental Groups

All animal handling and experimental procedures complied with protocols approved by Kobay DHL (Date: 25.01.2019 and Issue: 336) and the Hacettepe University Institutional Animal Care and Use Committee (Date: 20.04.2020 and Issue: 2020/03-05), following NIH animal welfare guidelines. This study utilized 30 female New Zealand White rabbits, aged 10–12 weeks and weighing between 1.9 and 2.4 kg. All graft materials were washed with PBS containing 1% P/S.

The rabbits were randomly divided into three groups. In the first group, 5 mm muscle pieces were removed from the attachment sites of the vertical rectus muscles (superior and inferior recti) in the right eye. Decellularized muscle grafts (dEOM) of the same size were sutured into the defect using 7/0 polyglactin sutures. The left vertical rectus muscle in this group served as a positive (+) control, visualized without any defect.

In the second group, an aPCL–PLGA graft was used for the muscle defect in the right eye, while a muscle piece from the right eye was used as an autograft in the left eye’s vertical rectus.

In the third group, a *g*-dEOM/aPCL–PLGA graft was employed for the muscle defect in the right eye, and a PLGA graft served as a negative (−) control in the left eye (Figure 1).

Defects and grafts were evaluated and imaged 15- and 45-days postsurgery. Clinical examination scores ranged from 0 to +3 based on parameters such as secretion, conjunctival hyperemia, episcleral and scleral vascularization, adhesions between the conjunctiva and muscle, adhesions between the sclera and muscle, graft distance from the cornea, and graft width. All procedures were conducted under anesthesia as previously described. For histopathological analysis, 5 mm muscle samples were collected from the graft area of 15 rabbits at both 15- and 45-days postsurgery. Electrophysiological analysis (Figure S2) was performed on the superior rectus muscles on day 45.

#### 2.4.2 Histopathological Analysis and Immunohistochemistry

Muscle samples from each group were fixed in a 10% buffered formalin for 24 h at room temperature and embedded in paraffin to prevent tissue autolysis. Radial sections (3–4 slices) of 4 μm thickness were taken from the region between the primary and defective tissue regions in paraffin with a microtome and the area between the primary and defective tissue regions and stained with H&E and MTC.

For protein expression monitoring, tissue sections were dehydrated through a series of xylene and graded alcohols, then boiled in a microwave in 0.1 M citric acid solution. After washing three times for 5 min with PBS, sections were incubated in 3% hydrogen peroxidase for 20 min. Following another wash, sections were blocked with serum for 20 min. After blocking, sections were treated overnight with primary antibody solutions (MyoD 1:100, MyH 1:100, Pax7 1:100, Pitx2 1:100, Anti Dystrophin 1:300 dilution). After washing, they were incubated with biotinylated secondary antibody and streptavidin-labeled peroxidase, respectively, for 60 min. Peroxidase activity was visualized using DAB chromogen and counterstained with Mayer’s hematoxylin.

Protein expressions were evaluated under light microscopy with ImageJ software (USA). Comparative analyses in immunocytochemistry and intensity quantification were also conducted using ImageJ, with expression levels presented as mean ± standard deviation.

### 2.5 Statistical Analysis

Statistical analysis was conducted using GraphPad Prism 6 software (Graphpad, San Diego, CA) for *in vitro* analysis and SPSS (Statistical Package for Social Sciences Inc., Chicago, Illinois, ABD) 23.0 for *in vivo* analysis. Data between groups were analyzed for significance using one-way ANOVA. Statistical significance was defined as *p* < 0.05.

## 3 Results and Discussion

### 3.1 Decellularization of Bovine Extraocular Muscles and Characterization

The decellularization protocol yielded white, elastic muscle tissue pieces with high strength, suitable for suturing, aligning with existing literature (Figure 2A).^20,28^ SEM images revealed that the morphological properties of decellularized muscle tissue and native muscle tissue (nEOM) were similar, including fiber structures and porosity. While nucleated cell structures were visible in nEOM with H&E staining, no cellular presence was observed in the dEOM groups, as expected. DAPI staining of nEOMs showed dark-blue stained cell nuclei, while dEOMs displayed only nonspecific fluorescent staining. Furthermore, the preservation of connective tissue in decellularized bovine eye muscle samples was confirmed through MTC staining (Figure 2A).

**Figure 2:**
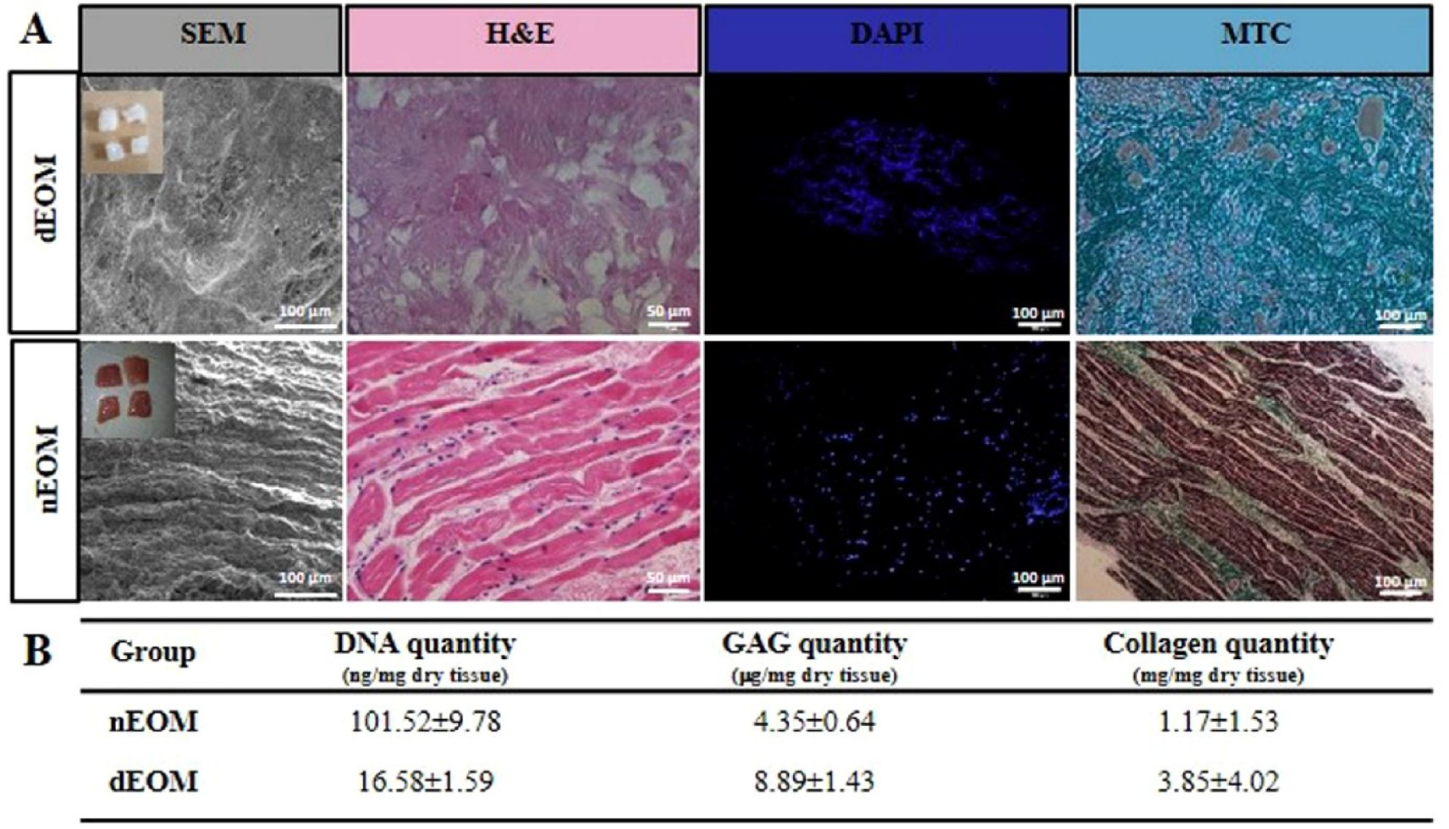
Comparison of native extraocular muscle (nEOM) and decellularized extraocular muscle (dEOM). (A) SEM (100 μm), H&E (50 μm), DAPI (100 μm), and MTC (100 μm) staining images of the muscles, from left to right, respectively. (B) Summary of double-strand DNA, GAG, and collagen contents in the muscles (*n* = 3). The upper left images show camera photos of the extraocular muscles.

The aim of obtaining ECM through decellularization is to preserve mechanical integrity, the three-dimensional tissue structure, and biological activity while effectively eliminating cellular and nuclear contents.^29^ Successful decellularization was confirmed by measuring the amount of double-stranded DNA, which was found to be less than 50 ng/mg dry tissue (Figure 2B). Additionally, histological analysis showed no visible nuclei in the dEOMs when examined with H&E and DAPI staining.^20^

Collagen and glycosaminoglycan (GAG) contents, which are crucial for maintaining ECM integrity, were found to be comparable in dEOMs and nEOMs (Figure 2B). The retention of hydroxyproline and GAG levels in the decellularized tissues suggests effective preservation of collagen and overall tissue structure, consistent with findings from other studies.^30,31^

In our study, higher levels of GAG and collagen in dEOMs compared to nEOMs may be attributed to the increased concentration of these molecules in the cell-free ECM, supporting the preservation of their structures. The relatively small size of the prepared tissues (10 × 10 × 1.5 mm^3^) likely facilitated a shorter decellularization period (approximately 5–6 days), allowing for better retention of collagen and GAG. In contrast, thicker tissue samples required longer exposure to SDS (approximately 9–10 days) to achieve complete whitening and remove all cellular content.

### 3.2 Fabrication and Characterization of aPCL–PLGA and *g*-dEOM/aPCL–PLGA Membranes

#### 3.2.1 Fabrication of aPCL–PLGA Membranes

Poly­(caprolactone) (PCL), a synthetic and biodegradable polymer approved by the FDA, is widely utilized in both soft and hard tissue applications, particularly in muscle tissue engineering.^22,32^ PCL offers significant advantages, including structural support, conductivity, and solubility in various solvents.^22,33^ Various fabrication methods, such as solvent casting-particle extraction, electrospinning, melt deposition modeling, and 3D printing, can be employed to create PCL scaffolds. Among these, electrospinning is particularly valued for producing micro/nanofiber matrices. However, achieving the desired thickness for nanofibrous scaffolds requires prolonged electrospinning, which can destabilize the spinning solution. Moreover, PCL degrades over extended periods, leading researchers to explore combinations of PCL with gelatin^34^ and collagen^35^ to enhance *in vitro* muscle tissue formation.

In our study, the membrane component of the tissue grafts (aPCL–PLGA) was created by collecting PCL nanofibers on PLGA membranes. PLGA’s shorter degradation time and better light transmittance, due to its transparent structure postsolvent casting, make it a suitable choice.

Topography and bioactivity are crucial in designing tissue scaffolds for eye muscle applications, as they facilitate cell adhesion. These properties should be tailored to the target cell and tissue characteristics. Since striated skeletal muscle comprises fiber bundles, it is essential to ensure that fibers are topographically aligned with appropriate diameter distribution and porosity. Additionally, scaffolds should incorporate suitable signaling molecules for enhanced bioactivity.^36^ Therefore, this study aimed to develop an aligned array of electrospun PCL nanofibers as a topographic signal to promote adhesion and differentiation of muscle tissue cells. Various electrospinning parameters were evaluated for optimal fiber alignment (Table S1), ultimately determining that the ideal values for voltage, solution flow rate, and syringe tip-collector distance were 11 kV, 0.25 mL/h, and 11 cm, respectively. The 2D fast Fourier transform (FFT) analysis indicated aligned fiber formation in the spindle view, while the circular view displayed irregular fiber formation.^37^ Aligned PCL fibers were observed in both the 2D FFT analysis and SEM images (Figure S1A–C). The average orientation/alignment angle was calculated from SEM images using the ImageJ/Fiji (USA) software and normalized to 90°. Approximately 80% of the aligned nanofibers fell within the ±20° range, indicating a high degree of alignment (Figure S1D). Furthermore, the goodness-of-fit value was determined to be 0.80, where a value of 1 represents perfect conformity to a Gaussian distribution and a value of 0 indicates complete deviation.

#### 3.2.2 Fabrication of *g*-dEOM/aPCL–PLGA Grafts

The *g*-dEOM/aPCL–PLGA grafts were created by gelling powdered ECM and depositing it onto the aPCL–PLGA membranes. SEM images clearly show a dense, homogeneous coating of ECM with a fibrillar structure on the PCL fibers (Figure 3A). Both PCL and PLGA contain carbon, hydrogen, and oxygen elements in their structure (Figure S3). It is anticipated that amine groups, and thus nitrogen atoms from proteins in the ECM, will be present in the gel-coated structure. Figure 3A shows that nitrogen atoms were detected in the *g*-dEOM/aPCL–PLGA membranes through SEM-EDX analysis, confirming the successful ECM coating.

**Figure 3:**
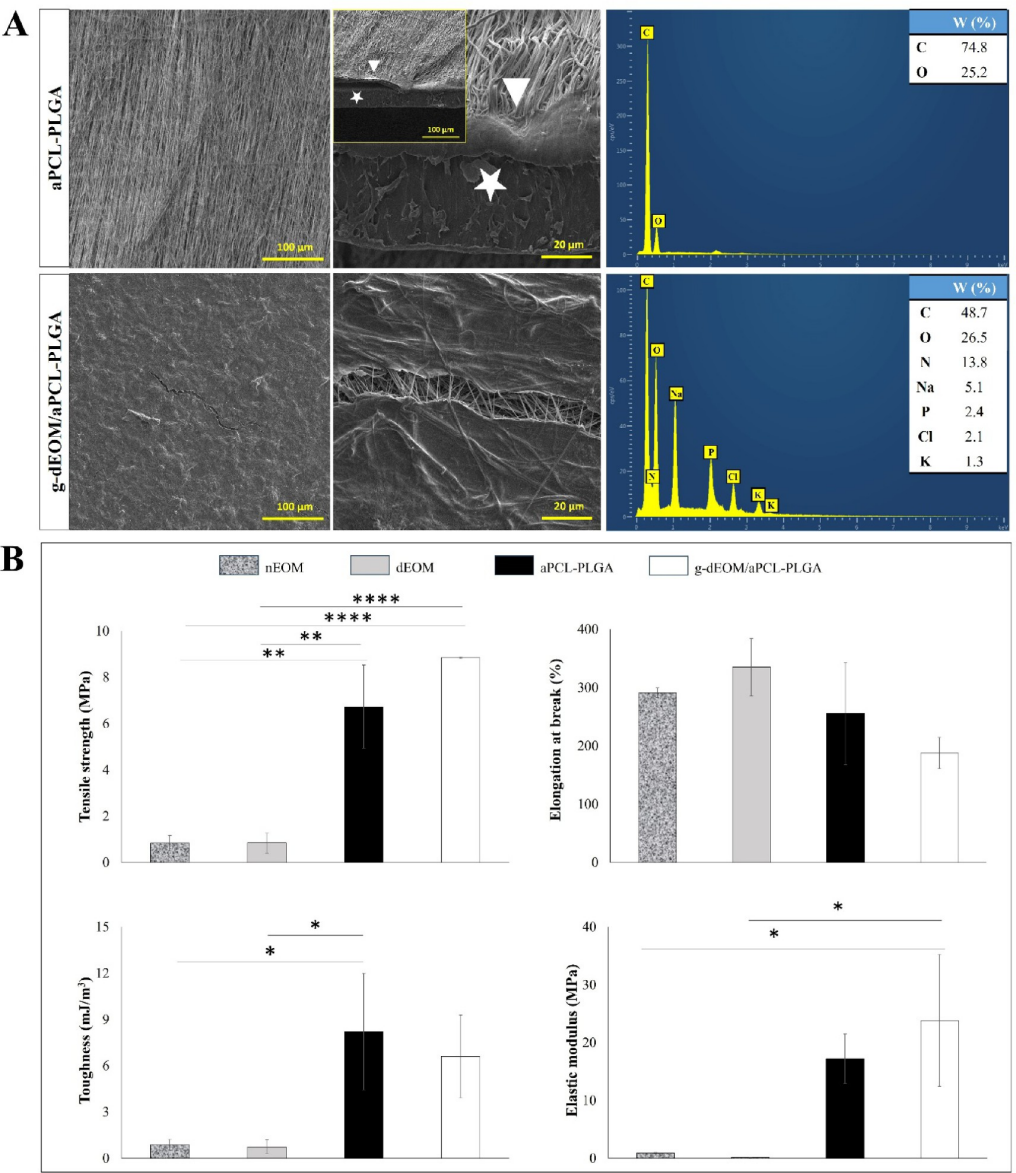
Morphological, chemical and mechanical analysis results of the grafts. (A) Comparison of membranes with and without ECM gel coating. Left, SEM images of membranes. The white star and arrow indicate the PLGA membrane and PCL nanofibers, respectively. The ECM structure is clearly distinguishable on the *g*-dEOM/aPCL–PLGA membranes. Right, EDX analysis results. The EDX analysis of *g*-dEOM/aPCL–PLGA shows the presence of nitrogen due to the ECM, unlike aPCL–PLGA. (B) Tensile test results of the grafts.

According to SEM images, the PLGA membrane has a thickness of approximately 0.045 mm, which increases to 0.065 mm with the addition of aPCL fibers. This thickness range for synthetic grafts was selected based on findings from previous studies in ocular tissue engineering.^38,39^ When designing graft materials, choosing the appropriate thickness is essential, as increasing the thickness of nanofibrous structures enhances mechanical properties but also results in more material that must be degraded and removed during muscle tissue regeneration. Additionally, rabbit eye muscle, like human eye muscle, is a delicate tissue composed of thin, microscopic fibrils, but it has a macroscopically sheet-like structure (approximately 0.5 mm thick). The aPCL–PLGA structure has demonstrated the required strength during muscle contraction, with no ruptures observed in any of the samples.

#### 3.2.3 Water Contact Angles

The water contact angles for the PLGA and PCL membranes produced via solvent casting were measured at 81.05 ± 7.94° and 102.45 ± 5.46°, respectively. For the *g*-dEOM/aPCL–PLGA groups, the contact angle was significantly lower at 35.00 ± 14.14°, while the aPCL–PLGA groups exhibited a contact angle of 95.90 ± 4.00°. As anticipated, the ECM-coated groups showed lower contact angles due to the dense protein structure present. This indicates that the ECM coating enhances the hydrophilicity of the aPCL–PLGA membrane, which is expected to promote cell adhesion.^40^

#### 3.2.4 Mechanical Strength

The mechanical properties of graft materials significantly influence the myogenic potential of muscle progenitor cells.^41^ Flexibility in grafts is functionally preferred during the early stages of myoblast transformation into striated muscle, similar to the properties of normal muscle tissue.^42^ As shown in Figure 3B, the toughness, elongation at break, and tensile strength of the decellularized muscle were comparable to those of normal muscle tissue, while the elastic modulus was lower. Literature indicates that the elastic modulus of nEOM collagen decreases when hydrated, allowing cross-linked hydrophilic materials to maintain their shape during *in vivo* processes.^43^

It has been noted that the elastic modulus in skeletal muscle can vary by location; for example, lower extremity muscles typically exhibit a higher modulus (ranging from 0.05 to 0.53 MPa) and collagen content compared to upper extremity muscles.^44^ In a study on the latissimus dorsi muscle of mice, the elastic modulus (approximately 0.5–0.6 MPa) remained largely unchanged postdecellularization with Triton-X and SDS.^45^ Our findings indicate no significant changes in tensile strength, elongation at break, or toughness following the decellularization of bovine EOM.

Titin proteins, typically found in eye muscles, are known to enhance muscle elastic modulus.^46^ Therefore, the removal of cellular content during decellularization is expected to reduce the titin protein structure, potentially decreasing the elastic modulus in decellularized muscle tissue. However, since extraocular muscles are not subjected to high forces compared to other striated muscles^47^ excessive deformation is not anticipated during normal conditions following suturing. In fact, decellularized muscle exhibits less deformation than normal tissue during surgical procedures, facilitating surgical manipulation and suturing.

When comparing the synthetic groups (aPCL–PLGA and *g*-dEOM/aPCL–PLGA), the elastic modulus values were similar, but an increase was noted in the presence of the ECM coating. This suggests that dEOM particles may agglomerate on the surface of PCL nanofibers, rendering the material less flexible. Notably, there was a significant difference between the mechanical properties of synthetic materials and those of natural tissues. While no specific studies have been conducted on graft development for EOM, our PCL–PLGA layered grafts were deemed to possess suitable mechanical properties for muscle regeneration, as corroborated by other muscle tissue studies.^26^

#### 3.2.5 Biodegradability Test

The biodegradability of PCL can range from months to years, depending on factors such as molecular weight, crystallinity, morphology, porosity, sample thickness, and environmental conditions.^48^ In contrast, PLGA typically degrades within approximately 2 months, with variations based on environmental pH and glycolic acid content.^49^ *In vitro* conditions showed about 20% mass loss in PLGA after 28 days, while PCL exhibited less than 5% mass loss.

Notably, the groups containing ECM (dEOM and *g*-dEOM/aPCL–PLGA) displayed faster degradation rates compared to aPCL–PLGA. This is attributed to the rapid dissipation of the ECM layer within the first 7 days, resulting in approximately 15% mass loss in dEOM over 28 days. The greater mass loss observed in *g*-dEOM/aPCL–PLGA (thickness ∼0.075 mm) compared to dEOM was due to the thicker bovine EOM used (approximately 1 mm). Additionally, the degradation profile of *g*-dEOM/aPCL–PLGA was found to be similar to that of aPCL–PLGA, likely due to its dense PLGA content.

After the onset of tissue regeneration in the first 4–5 days following muscle damage, a plateau is reached by the end of 2 weeks, which continues to decline until 3–4 weeks. Additionally, for the stability and mobility of rapidly contracting striated muscles, it is important that graft materials do not degrade too quickly. Upon examining the biodegradability results of the grafts prepared in this study, it is observed that the dEOM gel, which serves as a biosignal for stem cells, remains separated from the structure during the first 7 days when regeneration progresses with increasing momentum. Aligned PCL fibers, which play a topographic signaling role for myofiber differentiation, degrade over a longer period as anticipated. Although PLGA degrades faster than PCL, its role in the study is to support the fibers; therefore, its presence in the structure for the duration of the healing period (28 days) is sufficient.

### 3.3 Cell Culture Studies

Following the characterization studies, *in vitro* analysis of C2C12 cells was performed using dEOM (10 × 10 × 1 mm^3^), aPCL–PLGA (10 × 10 × 0.065 mm^3^), and *g*-dEOM/aPCL–PLGA (10 × 10 × 0.075 mm^3^) grafts.

#### 3.3.1 Cell Viability

Cell proliferation within the grafts was assessed using a mitochondrial activity assay (MTT) over a 14-day incubation period. The optical density values are presented in Figure 4A. All graft groups supported cell adhesion and proliferation effectively. Mitochondrial activity increased during the first 5 days across all groups but then stabilized in the aPCL–PLGA and *g*-dEOM/aPCL–PLGA grafts, likely due to cellular differentiation. SEM images of myotube formation, a key indicator of differentiation, corroborated this observation.

**Figure 4:**
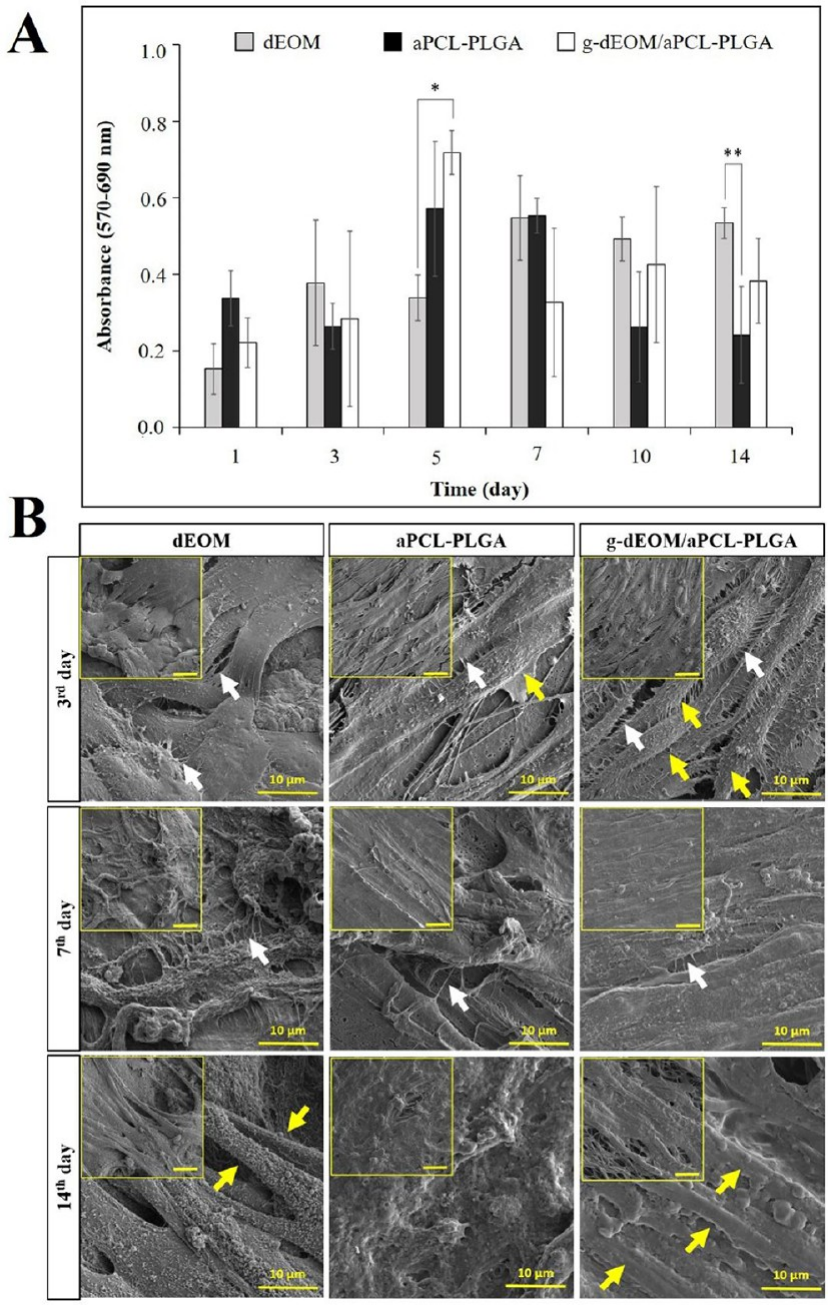
*In vitro* analysis with the C2C12 cell line on dEOM, aPCL–PLGA, and *g*-dEOM/aPCL–PLGA grafts. (A) MTT results, with statistically significant differences (*n* = 3), * p < 0.05, ** p < *0.01* when different groups were compared on the same day. (B) SEM images. Bar: 20 μm for small figures in the upper left. Yellow and white arrows indicate fused myotubes and neuromuscular junctions, respectively. Myoblast fusion and differentiation into myotubes were observed along the fiber direction in aPCL–PLGA and *g*-dEOM/aPCL–PLGA grafts.

When comparing mitochondrial activities daily, no statistically significant differences were observed between the groups, except on the fifth days (dEOM and *g*-dEOM/aPCL–PLGA) and 14th (dEOM and aPCL–PLGA) days. On the fifth day, a peak in mitochondrial activity was noted for cells on the *g*-dEOM/aPCL–PLGA surface (*p* < 0.05), whereas by the 14th day, the highest activity was associated with cells in the dEOM graft. However, this difference was not statistically significant when compared to the *g*-dEOM/aPCL–PLGA group. Moreover, no statistically significant differences were observed in the MTT assay results for *g*-dEOM/aPCL–PLGA grafts between days 5 and 7. Previous studies have shown that the *g*-dEOM coating method does not induce cytotoxic effects.^50,51^ In addition, SEM and confocal microscopy images obtained on day 7 (Figure 4B) revealed the formation of myotubes along the graft surface, indicating that cellular differentiation had occurred.

#### 3.3.2 Microscopic Imaging

To assess cell adhesion, proliferation, viability, and morphology, SEM analysis was conducted on days 3, 7, and 14 of the cell culture, complemented by confocal microscopy imaging on days 7 and 14. C2C12 myoblasts typically spread and proliferate upon attachment to the fiber surface, forming layers aligned parallel to the fiber axis. Previous studies have demonstrated that myoblasts enhance myotube formation during differentiation.^52,53^

As shown in Figure 4B, C2C12 cells developed filopodia consistent with the literature and exhibited a tendency to spread along the fibers. Myoblast fusion and differentiation into myotubes were observed in both aPCL–PLGA and *g*-dEOM/aPCL–PLGA grafts, with more pronounced myotube formation in the ECM-coated samples, particularly by day 3. Over the following days, all groups showed dense cellular coverage, with effective cell-to-cell communication facilitated by extensions formed between cells. These findings were consistent with the MTT results and SEM images.

Similar to the SEM images, confocal microscopy also revealed proper alignment of the fiber structures and cells in the dEOM, aPCL–PLGA, and particularly in the *g*-dEOM/aPCL–PLGA groups (Figure 5A). Cell counts were determined from DAPI-stained images acquired at 20× magnification using ImageJ (USA) software. To provide further clarity, the day 7 values of the aPCL–PLGA group were used as the control, and changes in cell number were expressed as fold increases (Figure S4). Examination of the confocal images showed that all three graft groups supported cell adhesion and proliferation; on days 7 and 14, the number of cells in the ECM-containing groups (dEOM and *g*-dEOM/aPCL–PLGA) was higher compared to the aPCL–PLGA group, with the difference reaching statistical significance on day 14 (Figure S4). The lower cell adhesion and proliferation observed on the aPCL–PLGA graft may be attributed to its hydrophobic surface (95.90 ± 4.00°), in contrast to the more hydrophilic *g*-dEOM/aPCL–PLGA graft (35.00 ± 14.14°). Cells on the aPCL–PLGA surface were occasionally observed with a rounded morphology (Figure 5A), further suggesting reduced attachment. Additionally, DAPI-stained images (Figure S4) demonstrated uniform nuclear staining intensity, indicating cell viability. Previous studies have shown that nuclei of nonviable cells exhibit brighter, whitish fluorescence, while viable cells display a distinct, vivid blue signal.^54,55^

**Figure 5:**
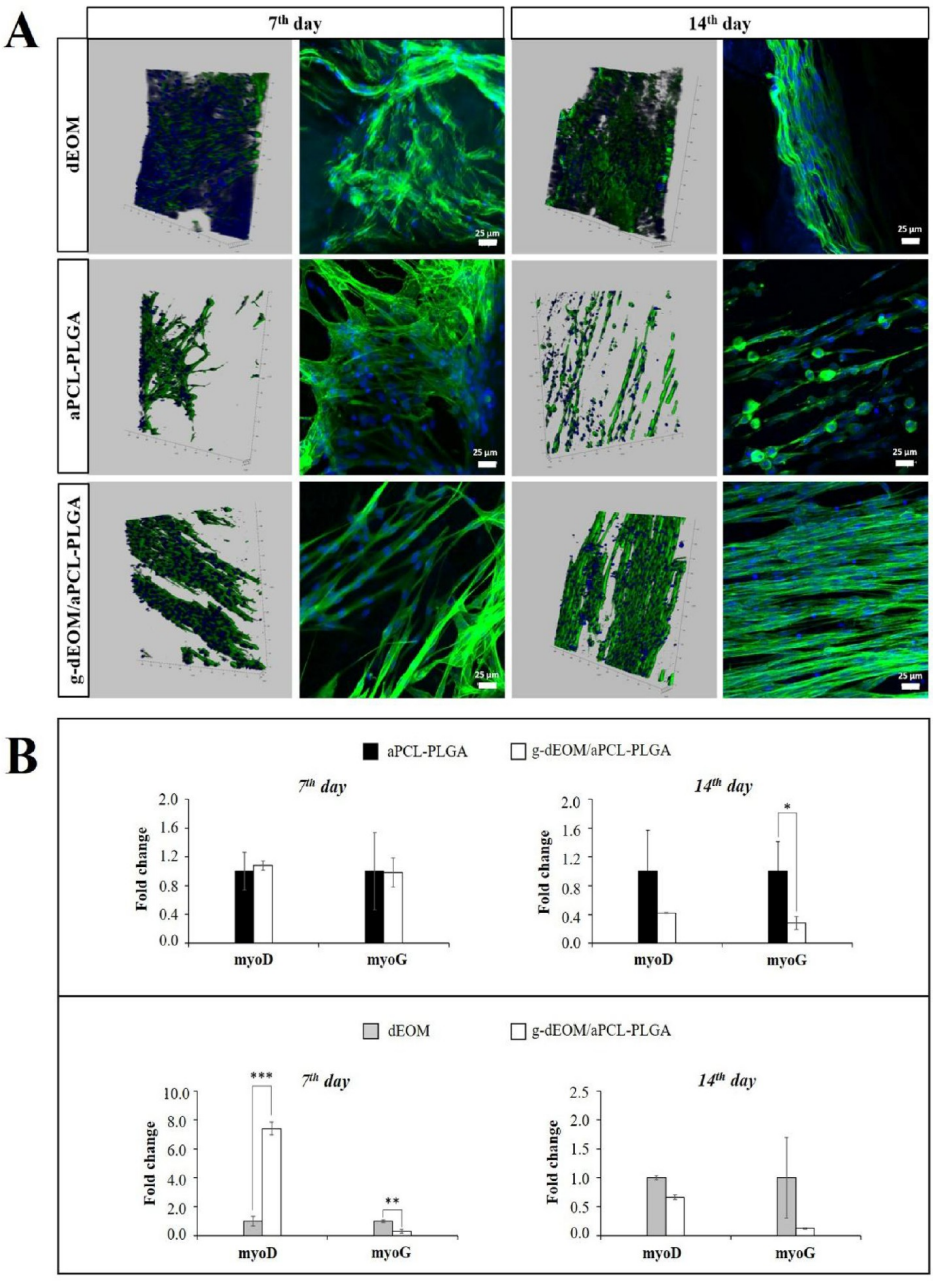
*In vitro* analysis with the C2C12 cell line on dEOM, aPCL–PLGA, and *g*-dEOM/aPCL–PLGA grafts. (A) Confocal microscopy images at different magnifications. The bar in the first image represents ∼100 μm depth along the *z*-axis. Green and blue fluorescence indicate F-actin filaments and cell nuclei, respectively. (B) RT-PCR results. The top row shows when *g*-dEOM/aPCL–PLGA is the control group, and the bottom row shows when dEOM is the control group. Statistically significant differences (*n* = 3), ^*^ *p* < 0.05, ^**^ *p* < 0.01, ^***^ *p* < 0.001.

#### 3.3.3 RT-PCR

Gene expression of MyoD and MyoG was compared between groups on days 7 and 14 through two approaches: first, by comparing aPCL–PLGA with the *g*-dEOM/aPCL–PLGA group as a control; second, by evaluating the *g*-dEOM/aPCL–PLGA group against the dEOM control group (Figure 5B). The rationale for these two evaluations stems from the differing characteristics of the groups.

MyoD expression is known to increase during the initiation of myogenesis and the recruitment of satellite cells into myofibers, indicating cell fusion. Similarly, MyoG is another marker of cell fusion that is upregulated during this process.^56^ While we anticipated an increase in MyoD and MyoG gene expressions as markers of muscle differentiation, contrary to our expectations, these genes remained nearly constant throughout the culture period.

Upon examining Figure 5B, no significant differences were observed between the groups overall. However, by the end of day 7, MyoD expression in the *g*-dEOM/aPCL–PLGA group was significantly higher than in the dEOM group (*p* < 0.001). This suggests that the ECM on the aligned fiber structure facilitated cell fusion. In contrast, by day 14, MyoG expression was found to be higher in the aPCL–PLGA group compared to the *g*-dEOM/aPCL–PLGA group (*p* < 0.05). This indicates that the effect of the ECM on differentiation was more pronounced within the first 7 days, while in the subsequent days, the cell proliferation mechanism may have become more dominant than differentiation.

### 3.4 *In Vivo* Animal Experiments

#### 3.4.1 Surgical Manipulations and Follow Up

In the *in vivo* experiments, six muscle groups were compared: dEOM, aPCL–PLGA, *g*-dEOM/aPCL–PLGA, (+) control, autograft, and (−) control (PLGA). The dEOM grafts were particularly well-suited for surgical manipulation due to their stability.

No infections or increased secretions were observed across all groups. Although the aPCL–PLGA grafts showed improved handling after being soaked in PBS with antibiotics, they were still not as amenable to surgical manipulation as the dEOM grafts. The autograft group, in contrast, was fragile and challenging to manipulate during suturing, making surgical procedures difficult due to the delicate nature of normal extraocular muscles.

#### 3.4.2 Early Healing Response

Representative images of the early healing response at day 15 are shown in the first row of Figure 6A. No significant secretions or infections were noted. The mean clinical scores for the parameters evaluated are summarized in Table 2.

**Figure 6:**
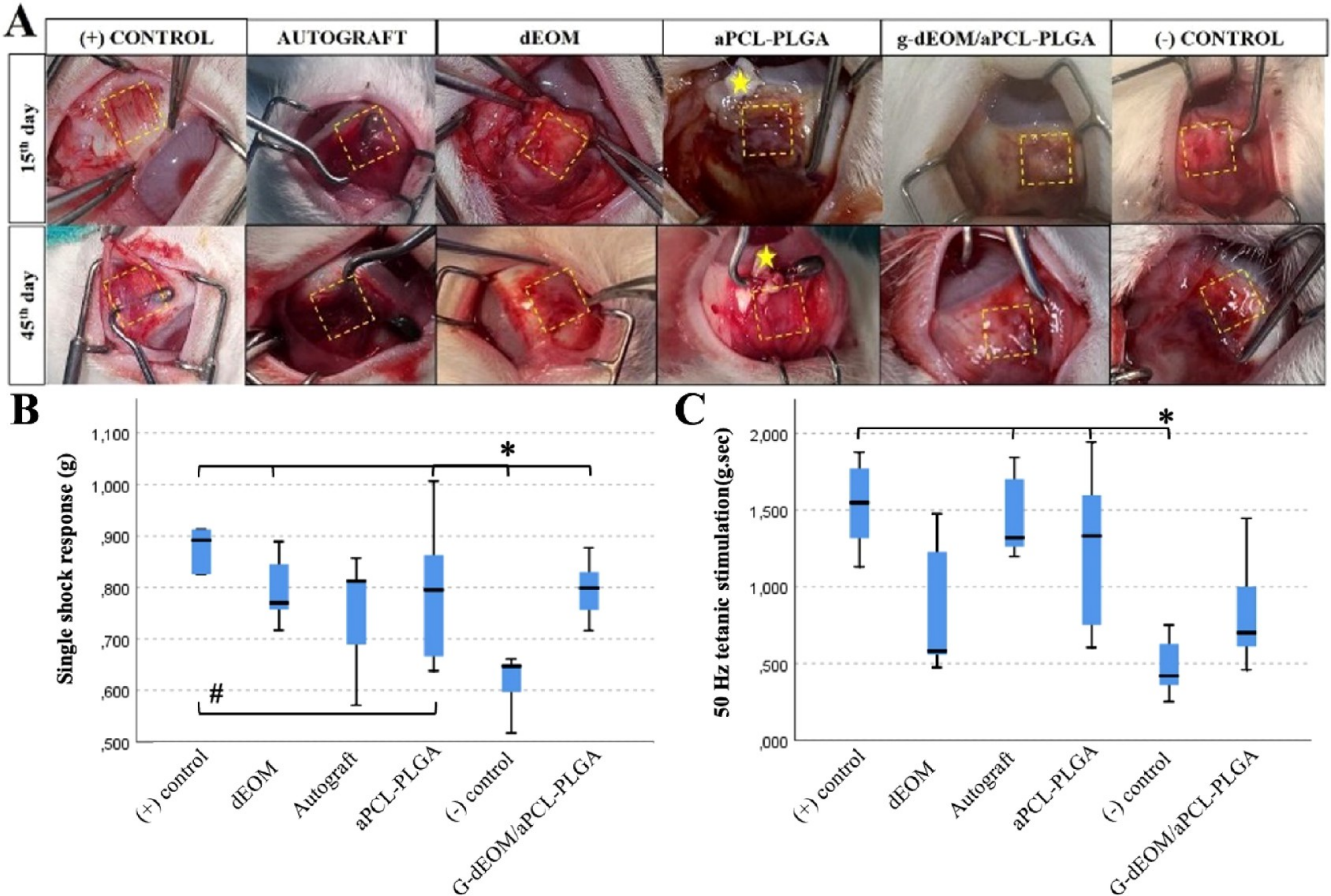
*In vivo* analysis with rabbits. (A) Macroscopic images of the grafts on days 15 and 45 following conjunctival incision. (B) Graph showing single twitch pulse responses (contraction amplitudes) from electrophysiological testing. *The (−) control group exhibited significantly lower amplitudes compared to all other groups, while the (+) control group showed significantly higher amplitudes than the aPCL–PLGA group (*p* < 0.05). (C) Graph depicting responses to 50 Hz tetanic stimulation. The (−) control group showed significantly lower responses than the other groups (*p* < 0.05). Rectangles highlight defect regions, while stars indicate areas of degenerated or residual graft material.

As expected, the (+) control group, which underwent no surgical intervention, exhibited the closest distance to the cornea. The distances in other groups were similar (*p* > 0.05). Some muscles receiving dEOM grafts exhibited edema and protrusion, which likely contributed to the increased distance of these grafts from the cornea, indicating instability due to early edema. The aPCL–PLGA grafts were observed to become opaque and calcified, with notable distortion and protrusion from the wound, as depicted in the first row of Figure 6A. Similar issues were observed in some PLGA and *g*-dEOM/aPCL–PLGA grafts, suggesting that the PLGA grafts degrade *in vivo* through initial opacification, hardening, and eventual disintegration.

The appearance of the grafts after conjunctival opening is clearly visible in the top row of Figure 6A, with muscle capsules surrounding the dEOM and *g*-dEOM/aPCL–PLGA grafts.

#### 3.4.3 Late Healing Response

Similar to the early phase, no late reactions or infections were noted. A closer distance of the grafts to the cornea indicated better stability and healing. Mean clinical scores for the late phase are summarized in Table 2.

Statistical analysis revealed a significant difference in the adhesion distance of the muscle to the cornea between the (+) control group and the dEOM (*p* = 0.035), *g*-dEOM/aPCL–PLGA (*p* = 0.000), and (−) control (*p* = 0.000) groups. Additional differences were observed between the autograft and (−) control (*p* = 0.000), autograft and *g*-dEOM/aPCL–PLGA (*p* = 0.005), and aPCL–PLGA and (−) control (*p* = 0.000), as detailed in Table S3.

The appearance of the grafts on day 45 after conjunctival opening is illustrated in the bottom row of Figure 6A. Notably, the aPCL–PLGA graft appeared calcified, opaque, and degenerated, while extensive adhesions were observed in the PLGA-grafted muscle.

Similar to the early phase, no late reactions or infections were noted. A closer distance of the grafts to the cornea indicated better stability and healing. Mean clinical scores for the late phase are summarized in Table 1.

**Table 1:** Clinical Scores and Muscle Measurement Results on Days 15 and 45 across All Groups (*n* = 5)^a^

	(+) Control	Autograft	dEOM	aPCL–PLGA	g-dEOM/aPCL–PLGA	(−) Control
**Inflammatory secretion** (0–3)	0.00 ± 0.00	0.33 ± 0.82	0.14 ± 0.38	0.17 ± 0.41	0.14 ± 0.38	0.0 ± 0.0
	*0.00 ± 0.00*	*0.00 ± 0.00*	*0.55 ± 0.69*	*0.25 ± 0.45*	*0.50 ± 0.80*	*0.38 ± 0.78*
**Conjunctival hyperemia** (0–3)	1.14 ± 0.38	1.33 ± 0.52	1.43 ± 0.53	1.33 ± 0.52	1.14 ± 0.38	1.14 ± 0.38
	*1.00 ± 0.00*	*0.75 ± 0.62*	*1.64 ± 0.50*	*1.17 ± 0.72*	*1.50 ± 0.52*	*1.25 ± 0.45*
**Superficial episcleral vascularization** (0–3)	1.29 ± 0.49	1.83 ± 0.75	1.86 ± 0.38	1.67 ± 0.52	1.29 ± 0.49	1.29 ± 0.49
	*1.00 ± 0.00*	*1.25 ± 0.45*	*2.00 ± 0.00*	*1.58 ± 0.51*	*1.83 ± 0.72*	*2.25 ± 0.62*
**Deep scleral vascularization** (0–3)	1.29 ± 0.49	2.33 ± 0.52	1.71 ± 0.49	2.17 ± 0.41	1.83 ± 0.38	2.14 ± 0.69
	*1.09 ± 0.30*	*1.58 ± 0.67*	*2.00 ± 0.00*	*1.75 ± 0.45*	*2.00 ± 0.60*	*2.42 ± 0.51*
**Adherence between muscle and conjunctiva** (0–3)	1.14 ± 0.38	2.50 ± 0.55	1.86 ± 0.38	1.50 ± 0.55	1.71 ± 0.49	2.29 ± 0.76
	*1.36 ± 0.50*	*1.50 ± 0.52*	*2.10 ± 0.74*	*1.45 ± 0.97*	*2.17 ± 0.72*	*2.42 ± 0.79*
**Adherence between muscle and sclera** (0–3)	1.00±0.00	2.33 ± 0.52	1.29 ± 0.76	2.50 ± 0.84	1.71 ± 0.95	2.50 ± 0.79
	*1.27 ± 0.47*	*1.58 ± 0.79*	*2.27 ± 0.47*	*1.83 ± 1.03*	*2.42 ± 0.67*	*2.50 ± 0.67*
**Muscle insertion to cornea distance (mm)**	1.71 ± 0.27	2.08 ± 0.20	2.33 ± 0.6	2.50 ± 0.45	2.29 ± 0.76	2.00 ± 0.58
	*2.00 ± 0.77*	*2.38 ± 0.93*	*3.59 ± 0.49*	*3.17 ± 0.56*	*4.33 ± 1.01*	*5.30 ± 1.36*
**Width of muscle (mm)**	5.21 ± 0.39	5.25 ± 0.42	5.64 ± 0.56	5.22 ± 0.41	4.71 ± 0.39	4.93 ± 0.35
	*5.23 ± 0.85*	*4.29 ± 0.78*	*4.73 ± 0.72*	*4.25 ± 0.68*	*5.13 ± 0.80*	*5.45 ± 0.76*

a Italicized values indicate results from day 45.

Conjunctival hyperemia and superficial vascularization were comparable across all groups (Table 1). Deep scleral vascularization and adhesions between the sclera and muscle were least pronounced in the (+) control group, while the (−) control group exhibited the most severe reactions. The aPCL–PLGA graft showed the least reaction and adhesion, followed by the dEOM and *g*-dEOM/aPCL–PLGA grafts, respectively. The distance from the graft to the cornea was closest in the (+) control group, followed by the *g*-dEOM/aPCL–PLGA, dEOM, and aPCL–PLGA groups, with the (−) control group showing the greatest distance. Small peripheral nerve grafts have been proposed in the literature to minimize donor site morbidity, but they can limit muscle contractility.^9^ Consequently, it has been suggested that muscle-like biomaterials in dogs should undergo freezing and thawing, similar to natural muscles.^10^ At the end of our study, the autograft tissue appeared pale, fibrotic, and adhered to the underlying sclera. Notably, adhesions formed at the temporal and nasal edges and along the sutured area, particularly where the dEOM and *g*-dEOM/aPCL–PLGA grafts were applied. While adhesion at the suture line is beneficial for muscle stability, adhesions along the edges may hinder contraction. Therefore, we implemented antifibrotic treatment strategies to enhance functional integration with the grafts.

#### 3.4.4 *In Situ* Electrophysiology Tests

Electrophysiological assessments were conducted on day 45, revealing significant differences in single twitch and 50 Hz tetanic responses between groups (Figure 6B,C). The (+) control group exhibited the strongest response, while a statistically significant difference was noted between the (−) control and aPCL–PLGA groups.

Various factors influence the contractile response to electrical stimulation. For accurate on-site measurements, it was essential to moisten the muscle with Krebs solution and position the force sensor relative to the muscle plane. Additionally, electrode placement on the muscle was critical, as dense fibrous reactions at the distal end of the graft could inhibit signal conduction and impair contractile responses.

Single twitch responses and 50 Hz tetanic responses varied among groups. These physiological tests might also be influenced by physiological differences among the rabbits. At higher frequencies, peripheral tissues could contribute to the electrical response, leading to similar responses across groups (Table S4).

Fibrosis and reduced fracture resistance have been reported in autografts used for muscle repair,^57^ indicating that even autografts, previously deemed ideal, may not possess optimal regenerative capacity. Furthermore, if muscle resection is unnecessary, donor site morbidity remains a significant drawback. Studies have shown that reduced responses to tetanic stimulation can persist for up to 90 days postmuscle injury, even when muscle fibers remain intact.^58^

#### 3.4.5 Histopathological Analysis

H&E staining of tissue sections taken 15 days postsurgery revealed increased vascularization, inflammatory cells, and eosinophilic-stained graft material in all groups except the (+) control (Figure 7). Notable inflammatory responses and vascularization were observed, particularly in some edematous dEOM grafts. The presence of edema in some dEOM grafts likely resulted from individual rabbit differences or graft inhomogeneity. By day 45, muscle fibers appeared better organized, with some graft material remaining. A decrease in vascularization and inflammatory cells was also noted. Muscle fibers in the positive control and autograft groups showed minimal inflammatory reaction and vascularization, while dEOM and *g*-dEOM/aPCL–PLGA grafts demonstrated better organization.

**Figure 7:**
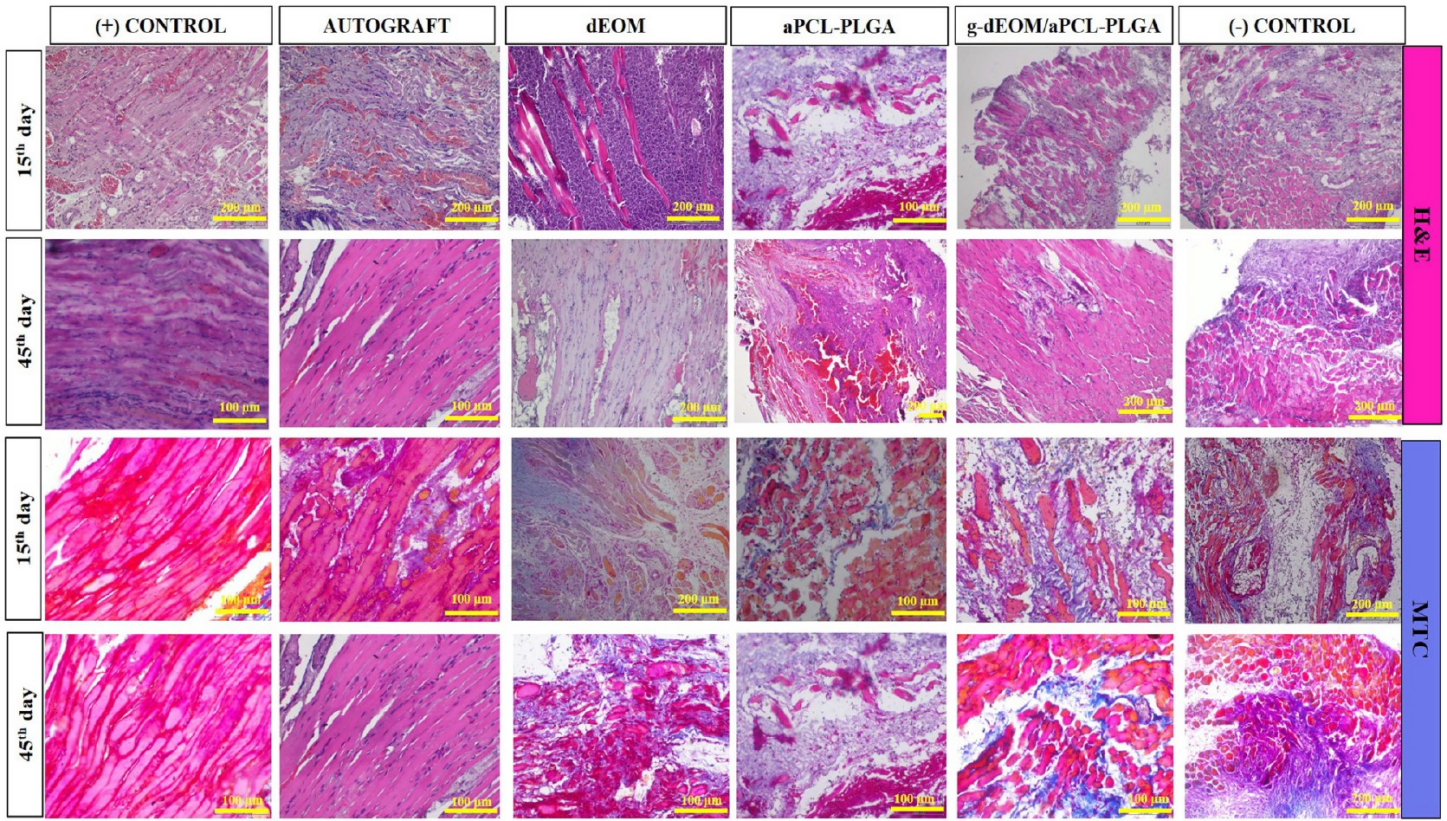
Histological staining images of all groups with H&E (top two lines) and MTC (bottom two lines) in the early (15th day) and late (45th day) phases of healing.

MTC staining revealed normal stromal collagen organization, similar to healthy muscle, in the control and autograft groups, while elastic fibers were observed in the (+) control. In contrast, the negative control group exhibited disorganized collagen organization throughout the study. While organized muscle fibers were apparent in the *g*-dEOM/aPCL–PLGA and dEOM groups in the late phase, they were less so in the aPCL–PLGA and (−) control groups. The inflammatory response in both early and late stages in the positive control and autograft groups likely reflected the influence of surgical procedures.

Longitudinal MTC slides showed elongation of muscle fibers toward the graft area in the dEOM, *g*-dEOM/aPCL–PLGA, and aPCL–PLGA groups. Sections displayed a fascicular arrangement of muscle fibers, immature muscle fibers, and myotubes interspersed with dense fibrous tissue. Similar findings in canine autografts have indicated a decrease in late-phase inflammatory cells alongside collagen-organized muscle fibers.^10^ Autografts harvested from the vertical rectus muscle of the fellow eye have also exhibited healing with fibrosis.^57^ Thus, some irregular muscle tissue and inflammatory cells in the sections are consistent with existing literature.

MTC-stained samples in the negative control and aPCL–PLGA groups displayed dense inflammatory cells and disorganized collagen structures, corroborating H&E findings. In the dEOM and *g*-dEOM/aPCL–PLGA groups, dense inflammatory cell infiltration was noted in the early phase, transitioning to uniformly arranged muscle fibers in the late phase. This alignment of clinical observations with microscopic evaluations reinforces the findings.

Immunohistochemical staining images from samples collected on day 45 postsurgery are summarized in Figure 8. Pax7, a transcription factor expressed in quiescent satellite cells and essential for skeletal muscle growth and repair, showed high expression in extraocular muscles.^7,59^ Numerous Pitx2-positive myonuclei were also observed, suggesting the recruitment of Pitx2-expressing precursor cells to existing muscle fibers during EOM myofiber remodeling—a normal process in adult muscle tissue.^60,61^ Both cytoplasmic (indicated by arrows) and nuclear (indicated by arrowheads) staining for Pax7 and Pitx2 were most prominent in the positive control group, followed closely by the autograft and dEOM groups. These findings indicate a greater regenerative potential in these groups compared to others.

**Figure 8:**
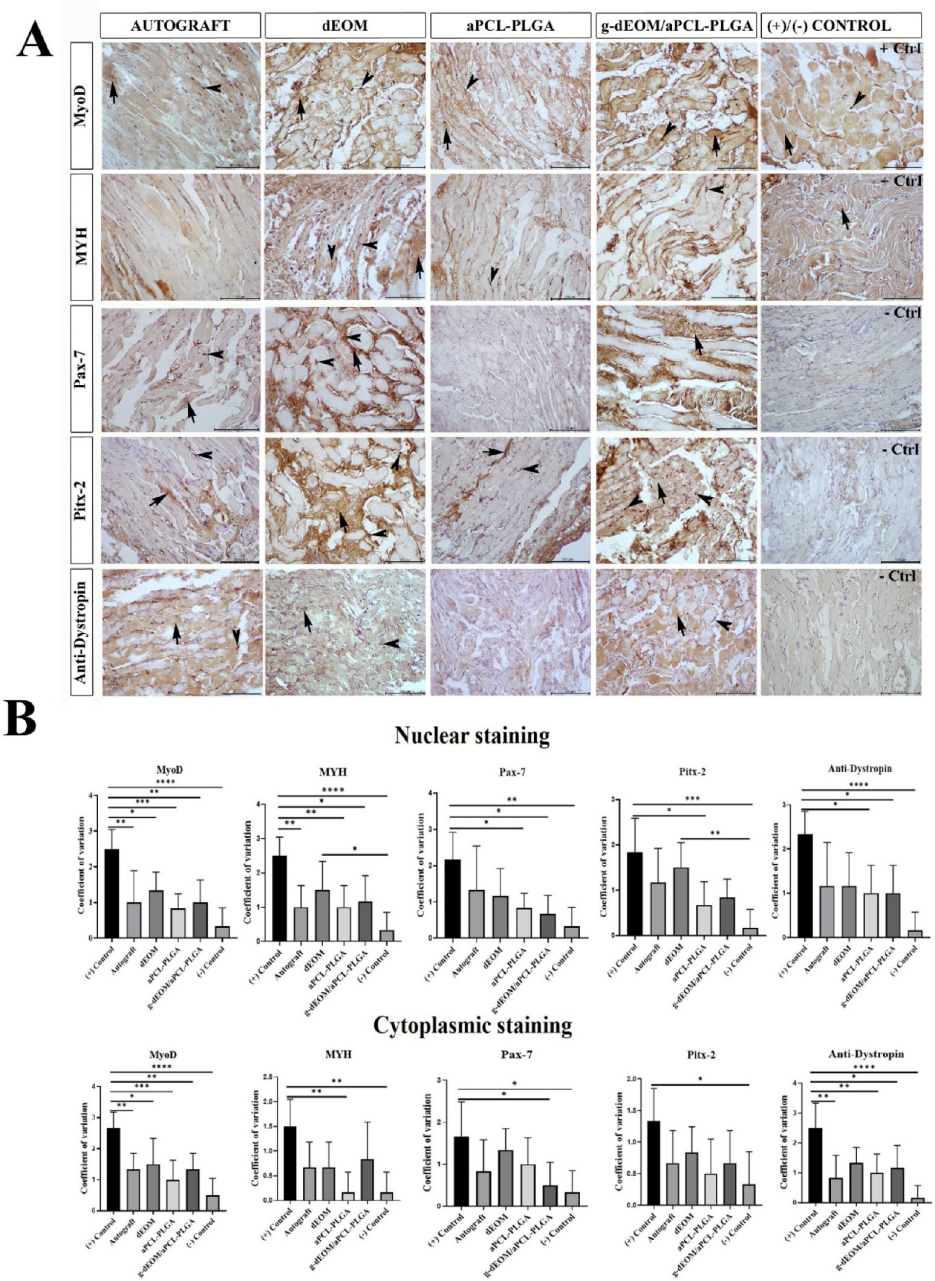
(A) Immunohistochemical staining showing the expression of MyoD, MYH, Pax-7, Pitx-2, and antidystrophin proteins in the tissue sections obtained on the 45th day of surgical procedure (Bar: 100 μm). Arrows: cytoplasmic staining, Arrowhead: nuclear staining. + Ctrl: Positive control, – Ctrl: Negative control. (B) The *p* values of statistical significance in comparisons of nuclear and cytoplasmic staining: (*n* = 5) * *p* < 0.05, ** *p* < 0.001, *** *p* < 0.0001.

The expression of myosin heavy chain (MHC) isoforms is essential for the contractile properties of skeletal muscle and also serves as a marker of muscle differentiation,^62^ alongside MyoD.^56^ In our study, nuclear MHC expression (indicated by arrowheads) was significantly higher in the dEOM graft group compared to the *g*-dEOM/aPCL–PLGA graft group (Figure 8). Although the differences were not statistically significant, both MHC and MyoD expression levels were elevated in the dEOM and *g*-dEOM/aPCL–PLGA grafts relative to the other groups.

Dystrophin is a structural protein that stabilizes muscle cell membranes and plays a crucial role in maintaining muscle function and integrity. In our study, higher immunoexpression of dystrophin was observed in both the autograft and dEOM graft groups compared to the *g*-dEOM/aPCL–PLGA graft group; however, these differences were not statistically significant (Figure 8A,B).

A comprehensive evaluation of the immunohistochemical findings indicates that the autograft group demonstrated the most pronounced muscle regeneration among all experimental groups, followed by the dEOM and dEOM/aPCL–PLGA graft groups. Nevertheless, the absence of statistically significant differences between the groups limits the ability to draw definitive conclusions.

Table 2 presents a comparative summary of the grafts fabricated in this study, along with their *in vitro* and *in vivo* performance characteristics. The obtained data suggest that both the dEOM and dEOM/aPCL–PLGA grafts hold potential for application in extraocular muscle regeneration.

**Table 2:** Comparison of Mechanical, Biological, and *In Vivo* Characterization of Grafts Fabricated in This Study (*n* = 3 for *In Vitro*, *n* = 5 for *In Vivo*)

	aPCL–PLGA	*g*-dEOM/aPCL–PLGA	dEOM
Elongation at break (%)	255.18 ± 87.37	187.45 ± 26.63	334.75 ± 49.37
Toughness (mJ/m^3^)	8.21 ± 3.78	6.61 ± 2.69	0.74 ± 0.47
Elastic modulus (MPa)	17.2 ± 4.30	23.80 ± 11.41	0.11 ± 0.04
Mass loss on the 7th day (%)	7.92 ± 1.84	22.90 ± 2.98	14.93 ± 10.57
Water contact angle (°)^a^	95.90 ± 4.00	35.00 ± 14.14	-
Myotube formation	++	++++	+
Cell attachment	+	++	+++
Ease of handling	++	++	+++
Muscle regeneration	+	+++	+++
Inflammation	++	+++	++++

a Water contact angle of dEOM tissue was not measured because of its irregular surface structure.**+**: very weak, **++**: weak, **+++**: moderate, **++++**: strong, **+++++**: very strong.

## 4 Conclusion

Effective regeneration of functional and healthy tissue in extraocular muscle pathologies requires advanced tissue engineering strategies. The decellularized bovine extraocular muscle (dEOM) and *g*-dEOM/aPCL–PLGA grafts developed and characterized in this study demonstrate their capacity to support myoblastic cell behavior both *in vitro* and *in vivo*. These grafts promote cell proliferation, migration, and organization, highlighting their potential in regenerative medicine. These findings provide a strong rationale for developing next-generation, ready-to-use, acellular therapeutic grafts designed to treat extensive extraocular muscle injuries and promote physiological muscle regeneration.

## Supplementary Material



## Notes

Alignment of PCL nanofibers (Figure S1); additional photograph showing electrical stimulation (Figure S2); FTIR spectra of materials (Figure S3); cell proliferation data derived from CLSM (Figure S4); electrospinning parameters for PCL nanofibers (Table S1); primer sequences used for RT-PCR (Table S2); post hoc analysis of groups (Table S3); contractile response in all groups (Table S4) (PDF)

## ∇

DWI—Leibniz Institute for Interactive Materials, 52074 Aachen, Germany
