# Supplementary material for: Engineered Extraocular Muscle with Decellularized Tissue and Synthetic Biodegradable Polymers: Design, Properties, and In Vivo Studies
Source: ACS Biomater Sci Eng. 2025 Aug 13;11(9):5482–97. doi: 10.1021/acsbiomaterials.5c00073 (PMC12421506; doi:10.1021/acsbiomaterials.5c00073)
Supplement: Supplementary file 1 [file ab5c00073_si_001.pdf]

SUPPORTING INFORMATION FOR PUBLICATION

ENGINEERED EXTRAOCULAR MUSCLE WITH DECELLULARIZED TISSUE AND SYNTHETIC BIODEGRADABLE POLYMERS:DESIGN, PROPERTIES, AND *IN-VIVO* STUDIES

Fatma Yülek [1,2], Özge Ekin Akdere[2*], Sena Koç Akbayrak[3,4], Menemşe Gümüşderelioğlu[2,3**], Sevil Çaylı[5], Ebru Alimoğulları[5], Ayşen Erdem[6], Meltem Tuncer[6], Özbeyen Atalay[6]

[1]Yıldırım Beyazıt University Faculty of Medicine, Department of Ophthalmology, 06031, Ankara, Turkey

[2]Hacettepe University, Department of Bioengineering, 06800, Ankara, Turkey

[3]Hacettepe University, Department of Chemical Engineering, 06800, Ankara, Turkey

[4]Hacettepe University, [1]Graduate School of Science and Engineering, 06800, Ankara, Turkey

[5]Yıldırım Beyazıt University Faculty of Medicine, Department of Histology, 06031, Ankara, Turkey

[6]Hacettepe University, Faculty of Medicine, Department of Physiology, 06230, Ankara, Turkey

**Present address: DWI - Leibniz Institute for Interactive Materials, 52074, Aachen, Germany*

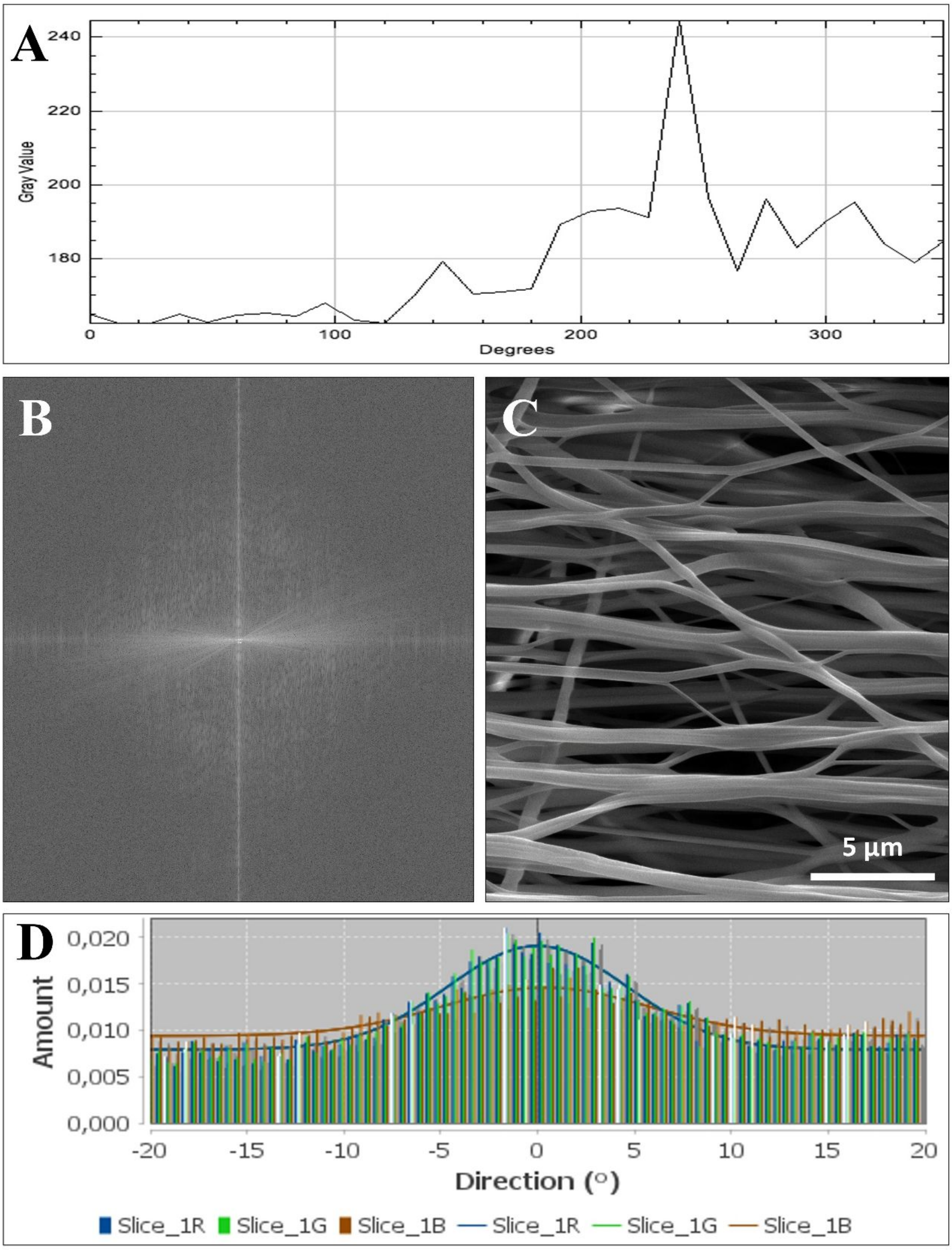

Figure S1. A) The 2D FFT approach image showing the aligned arrangement of PCL nanofibers coated on the PLGA membrane in the specified protocol (aPCL3) shows the main axis where most of the fibers are located. **B)** Image J Frequency shape. The presence of aligned fibers was determined by the spindle image. **C)** SEM image of the formed homogeneous fibers. **D)** Distribution of PCL nanofiber alignment normalized to 90°

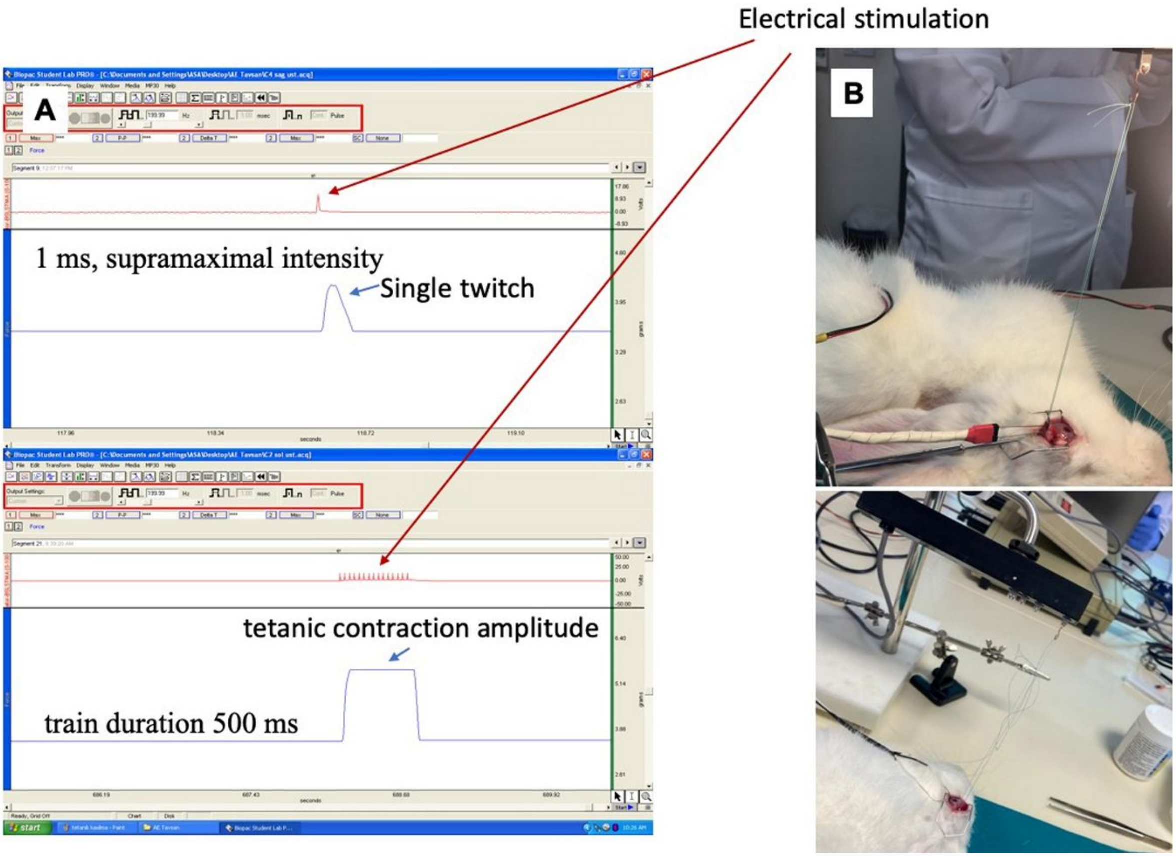

Figure S2. A) Example of contractile responses for single twitch and 50 Hz tetanic contractions. **B)** The angle of muscle and transducer was adjusted to a location of 90 degrees to the system in order to prevent power loss.

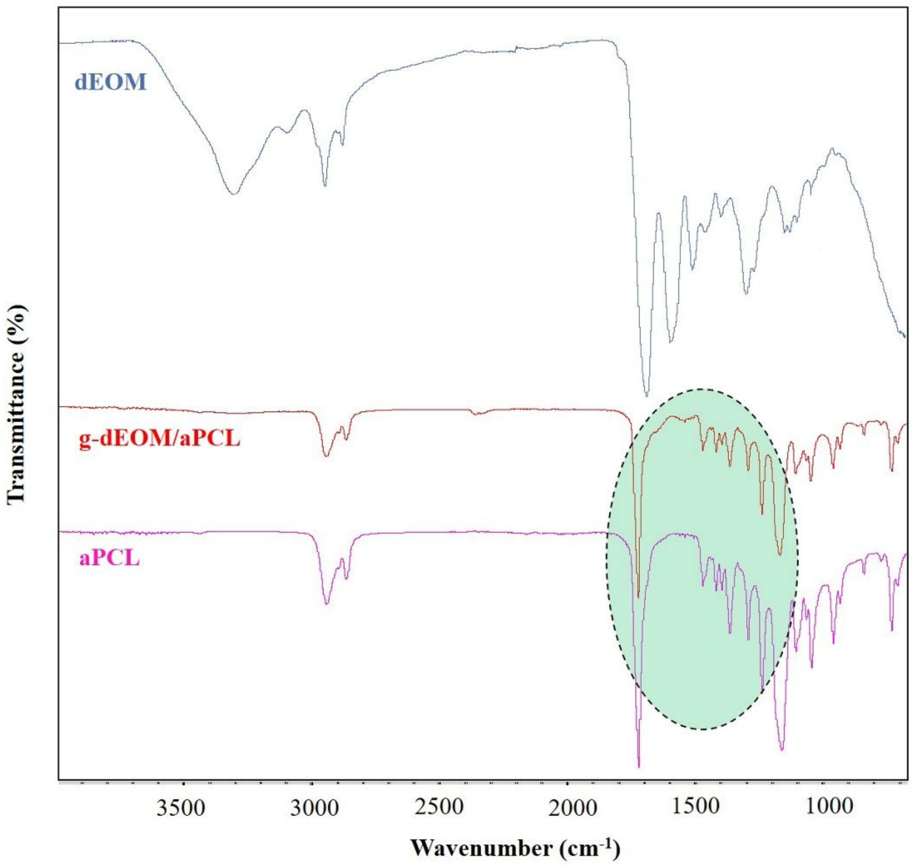

Figure S3. FTIR analysis results of ECM powder and PCL nanofibers. The green ellipse shows change of chemical bonding in presence of ECM in the PCL nanofiber (C-O elongation at 1660 cm^{-1} and N-H twist at 1540 cm^{-1} revealed by ECM addition).

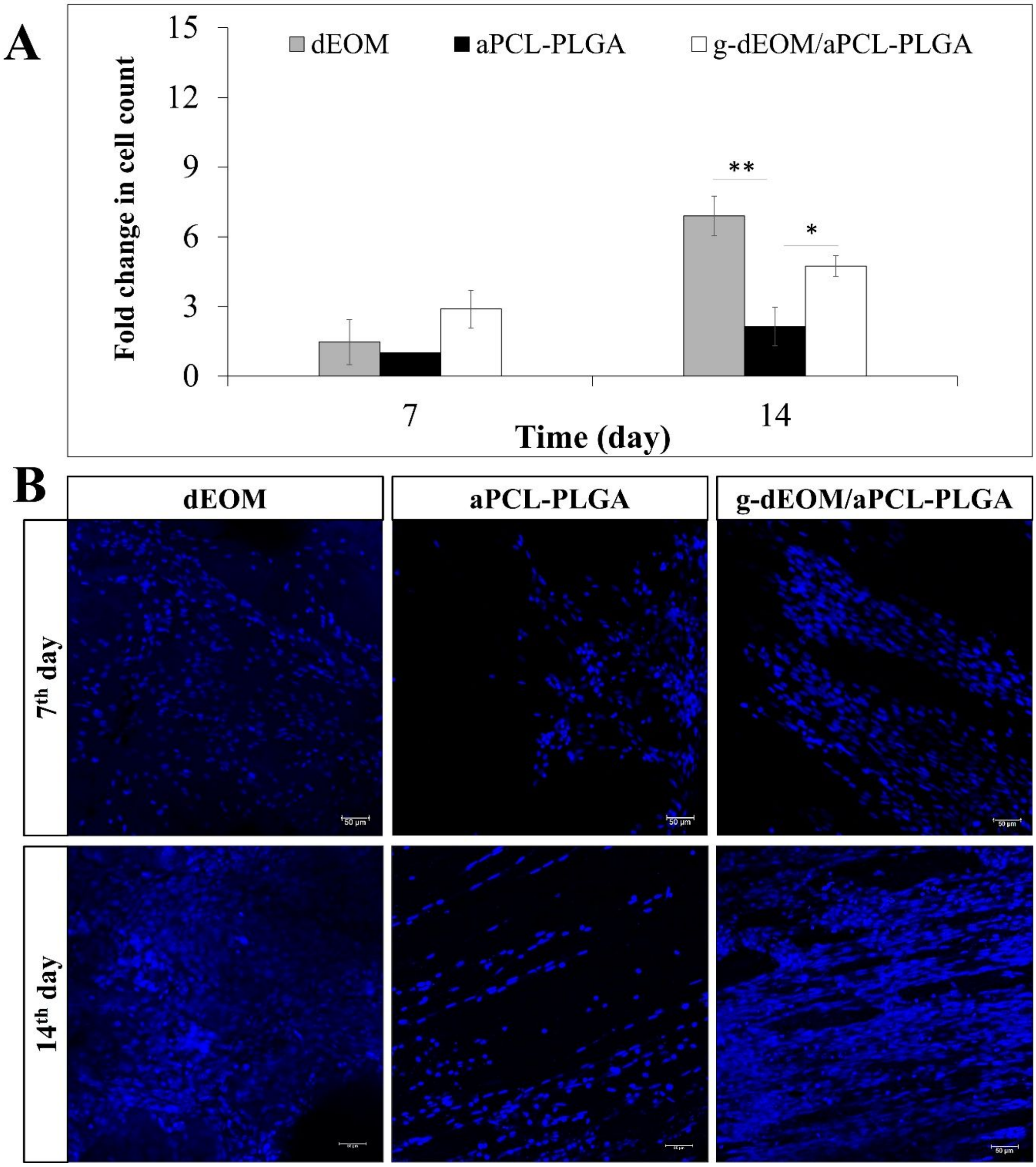

Figure S4. Cell proliferation data derived from confocal images of DAPI-stained cell nuclei. A) Measurement results obtained using Image J. B) Schematic representation of the images used for analysis. Statistically significant differences (n=3), * p<0.05, ** p<0.01.

Table S1. System parameters for obtaining aPCL nanofibers (n=3). Red shows the parameters optimized for the preparation of aligned nanofibers in aPCL-PLGA grafts.

Group	Flow rate (mL/h)	Voltage (kV)	Distance (cm)	Fiber diameter (nm)
aPCL1	0.25	14	11	398±176
aPCL2	0.25	14	15	511±285
**aPCL3**	**0.25**	**11**	**11**	**383±136**
aPCL4	0.25	11	15	481±257
aPCL5	0.50	14	11	555±248
aPCL6	0.50	14	15	395±205

Table S2. Primer sequences used for RT-PCR analysis.

Primer	Forward 5'-3'	Reverse3'-5'
**β-Aktin**	GCCCTGAGCCTCTTTTCCAG	TGCCACAGGATTCCATACCC
**MyoD**	CTGGTTCTTCACGCCCAAA	TAGGACAGACTTCTGCTCTTCC
**MyoG**	CCATGGTGCCCAGTGAAT	AAATGATCTCCTGGGTTGGG

Table S3. Post hoc analysis for groups with different muscle-corneal distances on the 45th day in all groups (n=5).

	p
**(+) CONTROL vs dEOM**	0.035
**(+) CONTROL vs g-dEOM/aPCL-PLGA**	0.000
**(+) CONTROL vs PLGA**	0.000
**AUTOGRAFT vs g-dEOM/aPCL-PLGA**	0.005
**AUTOGRAFT vs (-) CONTROL**	0.000
**aPCL-PLGA vs (-) CONTROL**	0.009

Table S4. Contractile response in all groups (n=5).

Group	Total twitch duration (ms)	Contraction amplitude (g)	Contraction index (50 Hz g.s)	Contraction index (75 Hz g.s)	Contraction index (100 Hz g.s)	Contraction index (150 Hz g.s)
**(+) CONTROL**	91±23.5	0.89±0.16	1.55±0.6	1.90±1.65	3.18±1.95	3.71±4.77
**AUTOGRAFT**	83±16.5	0.81±0.21	1.32±0.54	2.08±3.3	1.94±0.85	3.22±2.56
**dEOM**	85±23	0.77±0.13	0.58±0.83	2.05±1.46	2.00±2.52	2.99±4.48
**aPCL-PLGA**	85±15.5	0.79±0.28	1.33±1.09	2.16±1.95	2.54±3.56	3.56±3.8
**g-dEOM/aPCL-PLGA**	88±26.5	0.79±0.01	0.70±0.69	1.26±2.13	2.34±3.76	3.33±0.62
**(-) CONTROL**	81±26.5	0.65±0.09	0.42±0.38	1.01±0.86	1.27±1.03	1.88±1.46
